# Canonical Wnt signaling is antagonized by noncanonical Wnt5a in hepatocellular carcinoma cells

**DOI:** 10.1186/1476-4598-8-90

**Published:** 2009-10-22

**Authors:** Haluk Yuzugullu, Khemais Benhaj, Nuri Ozturk, Serif Senturk, Emine Celik, Asli Toylu, Nilgun Tasdemir, Mustafa Yilmaz, Esra Erdal, Kamil Can Akcali, Nese Atabey, Mehmet Ozturk

**Affiliations:** 1 Department of Molecular Biology and Genetics, Faculty of Science, Bilkent University, 06800, Ankara, Turkey; 2 Centre de Recherche INSERM-Université Joseph Fourrier U823, Institut Albert Bonniot, 38706 La Tronche Cedex, France; 3 Centre de Biotechnologie de Sfax, B.P "1177", 3038 Sfax, Tunisia; 4 Department of Medical Biology and Genetics, Dokuz Eylul University School of Medicine, Izmir, 35340 Turkey

## Abstract

**Background:**

β-catenin mutations that constitutively activate the canonical Wnt signaling have been observed in a subset of hepatocellular carcinomas (HCCs). These mutations are associated with chromosomal stability, low histological grade, low tumor invasion and better patient survival. We hypothesized that canonical Wnt signaling is selectively activated in well-differentiated, but repressed in poorly differentiated HCCs. To this aim, we characterized differentiation status of HCC cell lines and compared their expression status of Wnt pathway genes, and explored their activity of canonical Wnt signaling.

**Results:**

We classified human HCC cell lines into "well-differentiated" and "poorly differentiated" subtypes, based on the expression of hepatocyte lineage, epithelial and mesenchymal markers. Poorly differentiated cell lines lost epithelial and hepatocyte lineage markers, and overexpressed mesenchymal markers. Also, they were highly motile and invasive. We compared the expression of 45 Wnt pathway genes between two subtypes. TCF1 and TCF4 factors, and LRP5 and LRP6 co-receptors were ubiquitously expressed. Likewise, six Frizzled receptors, and canonical Wnt3 ligand were expressed in both subtypes. In contrast, canonical ligand Wnt8b and noncanonical ligands Wnt4, Wnt5a, Wnt5b and Wnt7b were expressed selectively in well- and poorly differentiated cell lines, respectively. Canonical Wnt signaling activity, as tested by a TCF reporter assay was detected in 80% of well-differentiated, contrary to 14% of poorly differentiated cell lines. TCF activity generated by ectopic mutant β-catenin was weak in poorly differentiated SNU449 cell line, suggesting a repressive mechanism. We tested Wnt5a as a candidate antagonist. It strongly inhibited canonical Wnt signaling that is activated by mutant β-catenin in HCC cell lines.

**Conclusion:**

Differential expression of Wnt ligands in HCC cells is associated with selective activation of canonical Wnt signaling in well-differentiated, and its repression in poorly differentiated cell lines. One potential mechanism of repression involved Wnt5a, acting as an antagonist of canonical Wnt signaling. Our observations support the hypothesis that Wnt pathway is selectively activated or repressed depending on differentiation status of HCC cells. We propose that canonical and noncanonical Wnt pathways have complementary roles in HCC, where the canonical signaling contributes to tumor initiation, and noncanonical signaling to tumor progression.

## Background

Hepatocellular carcinoma (HCC) is an epithelial cancer that originates from hepatocytes or their progenitors. It is the fifth most frequent neoplasm worldwide (>500,000 deaths/year), and its incidence is steadily increasing in the West [1]. Hepatocellular carcinoma is graded into four stages as well-differentiated, moderately differentiated, poorly differentiated and undifferentiated tumors, respectively. HCC arises as a very well differentiated cancer and proliferates with a stepwise process of dedifferentiation. Indeed, well-differentiated histology is exclusively seen in early stage and is rare in advanced HCC. Well-differentiated and moderately differentiated HCC cells are morphologically similar to hepatocytes, and are distinguished only by their smaller size and architectural organization as irregular trabecular or pseudoglandular patterns. In contrast, poorly differentiated and undifferentiated HCC cells are characterized with scanty cytoplasms and pleomorphism [2]. Like in other epithelial tumors, in HCC the progenitors evolve during tumor progression and become more and more autonomous. In this process, the tumor cells change their morphology and behavior; they loose cuboidal shape and polarity, and become more independent from neighboring tissues. Finally, they acquire the capacity to invade the underlying tissue and form distant metastases. These morphological changes are usually associated with progressive loss of biochemical and morphological features of hepatocytes, hence the process is qualified as "dedifferentiation" [3]. Portal venous invasion is significantly associated with poorly differentiated and undifferentiated HCCs and the tumor invasiveness is the most crucial factor in determining the long-term outcome for the patient [4].

Molecular changes involved in HCC dedifferentiation and invasiveness are known only partially. Epithelial markers such as hepatocyte nuclear factors and E-cadherin were reported to be down-regulated in HCC [3,5] and their loss is closely related to tumor invasion and metastasis [5]. In contrast, mesenchymal cell markers such as snail [6], twist [7] and vimentin [8] display positive correlation with HCC invasiveness and/or metastasis. These changes have been considered to represent the epithelial-mesenchymal transition (EMT) in HCC, based on in vitro studies [9-15]. Hepatocyte nuclear factor-4α (Hnf-4α) is essential for morphological and functional differentiation of hepatocytes [16,17], and its expression is downregulated during HCC progression in mice [18]. HCC dedifferentiation process is associated with a progressive accumulation of genomic changes including chromosomal gains and losses, as well as p53 mutations [19]. A rare exception to this picture is the status of the *CTNNB1 *gene that encodes β-catenin, a key component of the Wnt/β-catenin (canonical Wnt) signaling pathway.

Independent studies showed that β-catenin mutations are associated with a subset of low grade (well-differentiated) HCCs with a favorable prognosis and chromosome stability [20-25]. Among 366 unifocal HCCs studied by Hsu et al. [20], β-catenin mutations were associated with grade I histogoly. Another study with similarly high number of tumors (n = 372) also indicated that mutant nuclear β-catenin correlated positively with non-invasive tumor and inversely with portal vein tumor thrombi [23]. In addition, β-catenin mutations were associated with significantly better 5-year patient survival in these large cohorts. Direct study of canonical Wnt signaling activity in primary tumors is not possible. However, this can be studied indirectly by using target genes [22]. Using glutamine synthetase (encoded by canonical Wnt signaling target *GLUL *gene) as a sensitive and specific marker, Audard et al. [22] showed that 36% HCCs displayed canonical Wnt activation. These tumors exhibited significant features associated with well-differentiated morphology. The association of β-catenin mutation and nuclear translocation with well-differentiated tumor grade was also reported during hepatocellular carcinogenesis, using several transgenic mouse models [26]. Activation of β-catenin was most frequent in liver tumors from c-myc and c-myc/TGF-β1 transgenic mice. However, it was very rare in faster growing and histologically more aggressive HCCs developed in c-myc/TGF-α mice. Taken together, these studies suggest that nuclear translocation of β-catenin and activation of canonical Wnt signaling are early events in liver carcinogenesis, mostly affecting well-differentiated HCCs.

Mutations of β-catenin gene initially identified in colorectal cancers, cause constitutive activation of canonical Wnt signaling, as a result of aberrant β-catenin protein accumulation. Inactivating mutations of APC gene in colorectal cancer and AXIN1 in HCC also activate canonical Wnt signaling by the same mechanism. Therefore, tumors displaying β-catenin, APC or AXIN1 mutation are considered to display active or constitutive canonical Wnt signaling [27] Activation of canonical Wnt signaling appears to be a common event for colorectal cancer, as opposed to HCC with mutations limited to a subset of these cancers. Interestingly, transgenic mice expressing oncogenic β-catenin in hepatocytes develop only hepatomegaly [28,29], in contrast to intestinal polyposis and microadenoma when expressed in intestinal cells [30].

Taken together, published data indicate that Wnt pathway and β-catenin mutations may play a complex role in HCC. Close association of β-catenin mutation with low tumor grade suggests that canonical Wnt signaling has a dual role in HCC cells, depending on their differentiation state. As an initial attempt to characterize differentiation-dependent functions of Wnt pathway and canonical Wnt signaling in HCC, we used a panel of HCC-derived cell lines. We first performed gene expression and in vitro cell migration analyses to classify HCC cell lines into two distinct subtypes. The first subtype that we named here as "well-differentiated" was formed by epithelial cell lines with limited motility and invasiveness. Mesenchymal-like cell lines that have lost their epithelial-, hepatocyte-like features clustered into a second subtype named as "poorly differentiated". Next, we compared these two subtypes for the expression of 45 Wnt pathway genes, as well as for the activity of canonical Wnt signaling. Our findings provided evidence for the constitutive activation of canonical Wnt signaling in well-differentiated, but not in poorly differentiated cell lines. We also report the upregulation canonical Wnt3 ligand in the majority of HCC cell lines. Canonical Wnt8b was selectively expressed in well-differentiated cell lines. In contrast, noncanonical Wnt4, Wnt5a, Wnt5b and Wnt7b ligands were expressed selectively in poorly differentiated HCC cell lines. In addition, ectopic expression of noncanonical Wnt5a inhibited canonical Wnt signaling in two different cell lines. Our findings support the differential involvement of canonical and noncanonical Wnt signaling in HCC, depending on tumor cell differentiation state.

## Results

### Classification of hepatocellular carcinoma cell lines into "well-differentiated" and "poorly differentiated" subtypes

**Figure 1:**
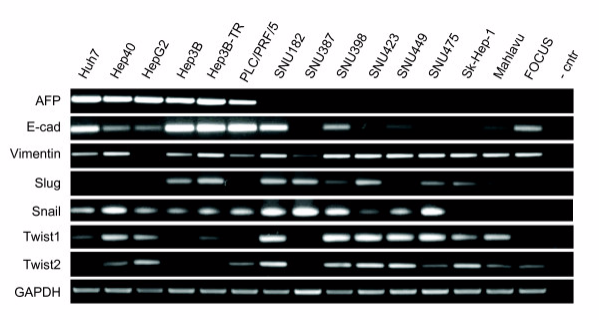
**Expression analysis of a-fetoprotein (AFP), E-cadherin and five mesenchymal cell markers in HCC cell lines**. Total RNAs were extracted from cell lines and used to detect gene expression by RT-PCR assay. GAPDH was used as a control for expression analyses shown here and in figures 4 to 7.

**Figure 2:**
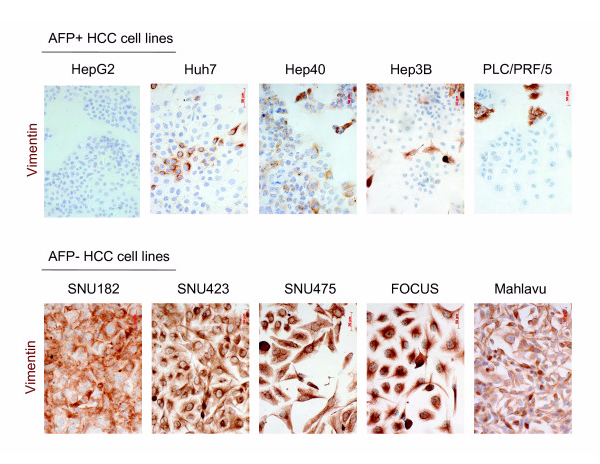
**Immunocytochemical analysis of mesenchymal marker vimentin protein in AFP+ and AFP- HCC cell lines**. Cells grown on coverslips were subjected to immunoperoxidase assay using anti-vimentin antibody (brown), and counterstained with hematoxylin (blue).

Fuchs et al. [12] have recently classified HCC cell lines into "epithelial" and "mesenchymal" types based on E-cadherin and vimentin expression. We performed a similar analysis using our cell line panel. Initially, we analyzed 15 cell lines (Figure 1). The expression of α-fetoprotein (AFP) was limited to six cell lines (Huh7, Hep40, HepG2, Hep3B, Hep3B-TR, PLC/PRF/5); the other cell lines being either not expressing (SNU182, SNU387, SNU398, SNU423, SNU449, SK-Hep1, Mahlavu, FOCUS) or weakly expressing (SNU475). All AFP-positive (AFP+) cell lines also expressed E-cadherin, whereas only 3/9 (33%) of AFP- cell lines expressed this epithelial marker. Mesenchymal cell markers including vimentin, slug, snail, twist-1 and twist-2 were also positive in most AFP- cell lines. These markers also displayed weakly positive expression in some AFP+ cell lines. For confirmation, we performed immunocytochemical analysis of vimentin protein expression in five AFP+ and five AFP- cell lines (Figure 2). We observed strong and homogenous immunostaining with all five AFP- cell lines. In contrast AFP+ cell lines were either negative or displayed heterogeneously positive immunoreactivity. These findings suggested that all AFP+ HCC cell lines were epithelial-like based on E-cadherin expression, but they also expressed some mesenchymal cell markers at variable degrees. In contrast, AFP- cell lines were usually negative for E-cadherin, and most of them were strongly positive for mesenchymal cell markers. The expression patterns of these mesenchymal markers showed marked heterogeneity. For example, SNU182 was positive for all five markers tested, whereas FOCUS was positive only for vimentin expression.

**Figure 3:**
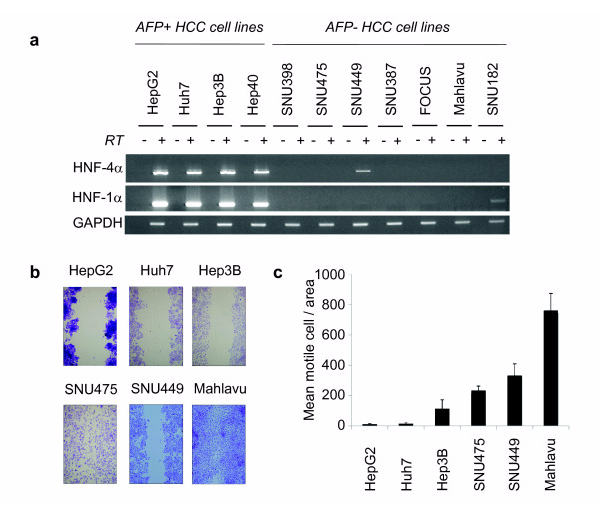
**Expression of hepatocyte lineage markers HNF-4α and HNF-1α in HCC cell line correlate with low motility**. (**a**)Selective expression of HNF-4α and HNF-1α in AFP+ HCC cell lines. Total RNAs were extracted from cells and used for RT-PCR analysis of HNF-4α and HNF-1α expression. GAPDH RT-PCR was used as a loading control. (**b. c**) Differential motility of AFP+ and AFP- HCC cell lines. Cells were cultured in six-well culture plates, and a single linear wound was made with a pipette tip in confluent monolayer cells. The distances between wound edges were measured at fixed points in each dish according to standardized template. After 24 hours migration, cell migration into the wound was visualized using phase contrast microscopy at ×20 magnification (**b**). The number of cells migrating beyond the wound edge was counted (**c**). Assays in six replicates, error bars; SD. SNU475 cells are larger cells giving rise to visual overestimation of migrating cell number in the picture shown in **b**.

We selected four AFP+, and seven AFP- cell lines for further analysis. HNF-4α and its downstream target HNF-1α are best known hepatocyte-associated epithelial cell markers [3]. These two genes that are involved in liver development and hepatocyte specification have previously been identified as specific markers for HCC cells with well-differentiated function and morphology [31]. The expression of these HNFs displayed perfect correlation with the expression of AFP (Figure 3a): four AFP+ cell lines (Huh7, Hep3B, HepG2, Hep40) were also highly positive for both HNF-4α and HNF-1α. In contrast, seven AFP- cell lines (SNU398, SNU475, SNU449, SNU387, FOCUS, Mahlavu, SNU182) did not express these factors. SNU449, another AFP- cell line displayed only weak HNF-4α expression. Epithelial cells including hepatocytes show low motility, in contrast to mesenchymal cells that display high motility and invasive behavior. To test whether epithelial and mesenchymal gene expression patterns of HCC cells correlated with their in vitro motility, we used wound-healing assay. After 24 hours of wounding, AFP- HCC cells (Mahlavu, SNU449, SNU475, SNU182) moved through the wound, whereas AFP+ HCC cells (Huh7, Hep3B, Hep G2, Hep40) cells did not (Figure 3b; Hep40 and SNU182 data not shown). A quantitative analysis of this data confirmed that poorly differentiated cell lines display higher motility (Figure 3c).

Based on expression analysis, together with in vitro motility and previously published invasiveness data, we classified our panel of HCC cell lines into two subtypes (Table 1). We qualified HepG2, Huh7, Hep3B and Hep40 as "well-differentiated" HCC cell lines, because they express AFP, E-cadherin, HNF-4α and HNF-1α, and they display low motility and/or low invasiveness. Most of these features are confined to "well-differentiated" HCC tumors ([2,18,32,33]. We qualified the remaining seven cell lines (SNU398, SNU475, SNU449, SNU387, FOCUS, Mahlavu, SNU182) as "poorly-differentiated" HCC cell lines, based on the lack of expression of both hepatocyte lineage and epithelial cell markers analyzed here. In addition, these poorly differentiated cell lines shared many features with mesenchymal cells including the expression of mesenchymal markers (vimentin, slug, snail, twist-1, and twist-2), high motility and invasiveness. These expression and migratory features are associated with tumor dedifferentiation and confined to poorly differentiated HCCs [6-8,14,34].

**Table 1:** Well-differentiated and poorly differentiated HCC cell lines according to hepatocyte lineage, epithelial and mesenchymal markers, and in vitro motility and invasiveness assays

**Cell Lines**	**Fetal Hepatocyte Marker**	**Epithelial & Hepatocyte Markers**			**Mesenchymal markers**					**Motility**	**Invasiveness**
	**AFP**	**HNF4a**	**HNF1a**	**E-cadherin**	**vimentin**	**slug**	**Snail**	**Twist-1**	**Twist-2**		

*Well-differentiated*											

HepG2	High	High	High	Low	(-)	Low	Low	Low	High	Low	Low [14]

Huh7	High	High	High	High	Low	(±)	Low	Low	(-)	Low	Low [14]

Hep3B	High	High	High	High	Low	High	Low	(-)	(-)	Low	Low ([74]

Hep40	High	High	High	Low	High	(-)	High	High	Low	Low	n.t.

*Poorly differentiated*											

SNU398	(-)	(-)	(-)	Low	High	Low	High	High	High	Low	n.t.

SNU475	(±)	(-)	(-)	(-)	High	High	High	High	Low	High	n.t.

SNU449	(-)	Low	(-)	(±)	High	Low	Low	High	High	High	n.t.

SNU387	(-)	(-)	(-)	(-)	Low	High	High	(-)	(±)	n.t.	n.t.

FOCUS	(-)	(-)	(-)	Low	High	Low	(-)	(-)	Low	n.t.	n.t.

Mahlavu	(-)	(-)	(-)	(±)	High	Low	(-)	High	Low	High	High [74]

SNU182	(-)	(-)	(±)	High	high	High	High	High	High	High	n.t.

(-); not detected; ±; traces; n.t.; not tested.

### Expression TCF/LEF family of transcription factors

**Figure 4:**
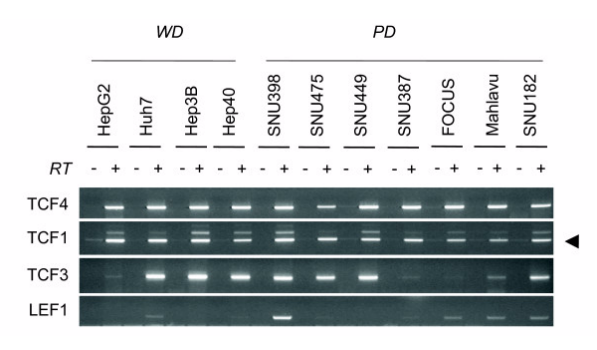
**Comparative analysis of TCF/LEF transcription factors in hepatocellular carcinoma cell lines**. Total RNAs were extracted from cell lines and used to detect gene expression by RT-PCR assay of four members of TCF/LEF family. See Figure 1 for GAPDH loading control.

Following the identification of well-differentiated and poorly differentiated HCC cell lines, we analyzed the expression of 45 Wnt pathway genes by RT-PCR assay. We first investigated the expression profile of TCF/LEF factors. The TCF-1 and TCF-4 were highly expressed in all HCC cell lines, while TCF-3 expression was limited to a subset of cell lines (Figure 4). LEF-1 transcript expression was weak, except for SNU398 cells. These findings indicated that at least two different nuclear factors mediating canonical Wnt signaling were expressed in any of HCC cell lines tested.

### Expression of Frizzled receptors and LRP co-receptors

**Figure 5:**
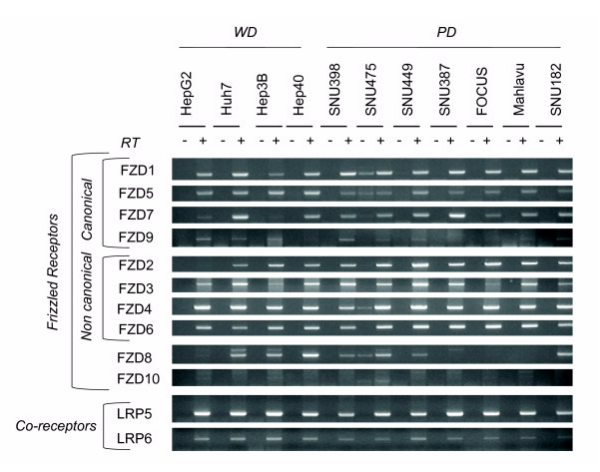
**Comparative analysis of Frizzled receptors and LRP co-receptors in hepatocellular carcinoma cell lines**. Frizzled receptors involved in canonical and noncanonical Wnt signaling were tested for expression by RT-PCR assay (Top). The expression of LRP co-receptors was analyzed similarly (bottom). Total RNAs were extracted from cell lines and used to detect gene expression by RT-PCR assay. See Figure 1 for GAPDH loading control.

Next we analyzed the expression of 10 Frizzled receptors and their two co-receptors. Two canonical (Fzd1, Fzd5) and three noncanonical (Fzd3, Fzd4, Fzd6) Frizzled receptors were expressed in all cell lines tested. Also, Fzd2, Fzd7 and Fzd8 were expressed in most cell lines independent of their differentiation status (Figure 5-top). Lrp-5 and Lrp-6 co-receptors also were consistently expressed in all cell lines (Figure 5-bottom). These findings indicated that HCC cell lines were equipped with the expression of several canonical and noncanonial Wnt signaling receptors, so that each HCC cell line was likely to respond to both canonical and noncanonical Wnt signals.

### Differential expression of canonical and noncanonical Wnt ligands

**Figure 6:**
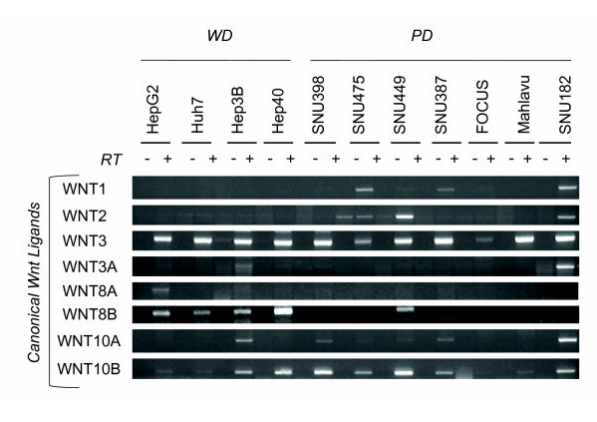
**Comparative analysis of canonical Wnt ligands in hepatocellular carcinoma cell lines**. Canonical Wnt ligands were tested for expression by RT-PCR assay. Total RNAs were extracted from cell lines and used to detect gene expression by RT-PCR assay. See Figure 1 for GAPDH loading control.

**Figure 7:**
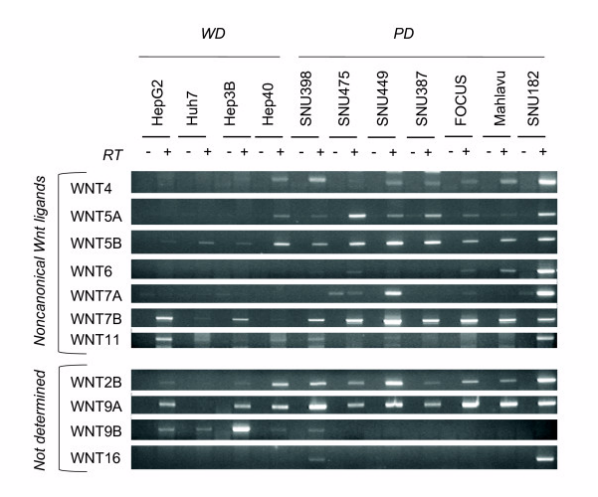
**Comparative analysis of noncanonical (top) and unclassified (bottom) Wnt ligands in hepatocellular carcinoma cell lines**. Noncanonical and unclassified Wnt ligands were tested for expression by RT-PCR assay. Total RNAs were extracted from cell lines and used to detect gene expression by RT-PCR assay. See Figure 1 for GAPDH loading control.

In humans, there are 19 known genes encoding canonical and noncanonical Wnt ligands [35]. We studied the expression profile of the complete list of human Wnt ligands (Figures 6 and 7). From the group of eight known canonical Wnt ligands, only Wnt3 was strongly and uniformly expressed in all cell lines tested. Wnt10b was also strongly expressed, but not in all cell lines (Figure 6). We observed selective expression of canonical Wnt8b in well differentiated cell lins. In contrast, among seven noncanonical Wnt ligands, Wnt4, Wnt5a, Wnt5b and Wnt7b were expressed in almost all poorly differentiated cell lines tested. This contrasted with their poor expression in well differentiated cell lines (Figure 7-top). Signaling specificity of four other Wnt ligands have not yet been clearly established [35]. Among these ligands, Wnt9a expression was detectable in nearly all cell lines tested. In contrast, Wnt9b and Wnt2b expressions were associated to well differentiated and poorly differentiated cell lines, respectively (Figure 7-bottom).

Our comprehensive analysis of Wnt signaling molecules in HCC cell lines revealed several features. First, with the exception of Wnt ligands, most of the major components of Wnt signaling pathway were expressed redundantly in HCC cell lines, independent of their differentiation status. In contrast, Wnt ligand expression displayed two types of selectivity. First, out of eight known canonical only Wnt3 and Wnt10b displayed strong expression in most cell lines, independent of differentiation status. Second, out of seven noncanonical Wnt ligands, four were expressed in HCC cell lines with a high selectivity for poorly differentiated ones. Well-diffferentiated cell lines displayed selective expression of Wnt8b. These findings may have several implications. Most, in not all HCC cell lines were equipped with an autocrine/paracrine canonical Wnt signaling system, as reported previously for breast and ovarian cancer cell lines [36]. In contrast, because of selective expression of noncanonical Wnt ligands, only poorly differentiated cell lines could serve from an autrocrine noncanonical Wnt signaling system. In addition, poorly differentiated HCC cells could also provide noncanonical Wnt signals to other cells by a paracrine mechanism. Finally, noncanonical Wnt ligands such as Wnt5a might inhibit canonical Wnt signaling in HCC cells, as previously reported in other cell types [37-39].

### Autocrine canonical Wnt signaling in well differentiated hepatocellular carcinoma cell lines

**Figure 8:**
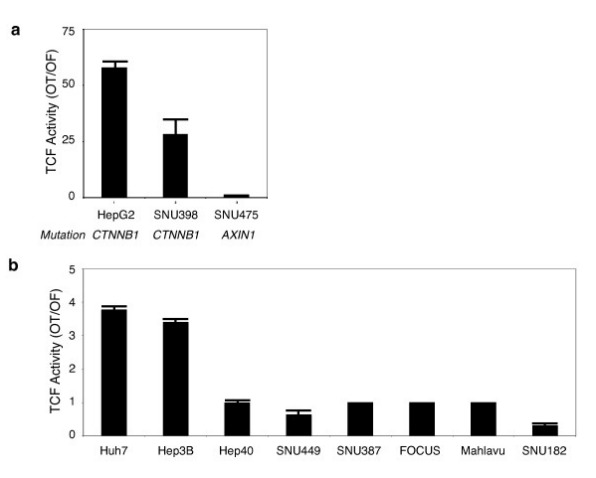
**Frequent constitutive activation of canonical Wnt signaling in well-differentiated, but not in poorly differentiated hepatocellular carcinoma cell lines**. (**a**) Comparative analysis of the canonical Wnt signaling in hepatoma cell lines with known mutations of β-catenin or Axin-1 genes. TCF reporter assay shows that well-differentiated HepG2 cells display high signaling activity. In contrast, canonical Wnt signaling is attenuated in poorly differentiated SNU398, and undetectable in poorly differentiated SNU475 cell line. Assays in triplicate, error bars; SD. (**b**) Comparative analysis of the canonical Wnt signaling in HCC cell lines with wild-type β-catenin and Axin-1 genes. Huh7 and Hep3B cell lines (both well-differentiated) display weak but significantly increased TCF reporter activity. Other cell lines (all poorly differentiated, except Hep40) display no detectable TCF reporter activity. TCF activity denotes the ratio of signals detected with pGL3-OT (OT) and pGL3-OF (OF) plasmids, respectively. Assays in triplicate, error bars; S. D. Cells were transfected with the reporter gene pGL3-OT (OT) harboring LEF-1/TCF binding sites for β-catenin and the corresponding pGL3-OF (OF) without these sites.

Canonical Wnt signaling activates TCF/LEF-dependent transcription, which can be monitored by reporters containing TCF/LEF-responsive elements [27,40]. We surveyed canonical Wnt signaling in HCC cell lines using TCF/LEF reporter pGL3-OT plasmid, as described previously [41]. First, we compared TCF/LEF (TCF) activity in three cell lines with known mutations in canonical Wnt signaling pathway (Figure 8a). Well-differentiated HepG2 cell line displays β-catenin mutation. Poorly-differentiated SNU398 and SNU475 cell lines display β-catenin and AXIN1 mutations, respectively [42,43]. Normalized TCF activity was the highest in HepG2 cells. Compared to HepG2, SNU398 cells displayed 50% less activity. More interestingly, despite a homozygous deletion leading to a loss of Axin1 expression (data not shown; [42,43], there was no detectable TCF activity in SNU475 cells. This contrasted sharply with another well-differentiated AXIN1 mutant HCC cell line, namely PLC/PRF/5 (Alexander) that displayed high TCF activity [42] (additional data not shown).

Next, we compared TCF activity of eight other cell lines that displayed wild-type β-catenin and AXIN1 status [41-43]. Hep40 cells that harbor a missense AXIN1 mutation/polymorphism (R454H) was included in this group, since functional significance of this mutation is unknown [41]. We detected weak, but significant (3-4 fold) TCF activity in well-differentiated Huh7 and Hep3B cell lines. On the other hand, all five poorly differentiated cell lines, as well as well-differentiated Hep40 cells displayed no detectable activity under our experimental conditions (Figure 8b).

Taken together, we collected TCF activity data from 12 HCC cell lines. Independent of β-catenin or AXIN1 status, TCF activity was detected in four out of five (80%) well-differentiated cell lines, whereas only one out of seven (14%) poorly differentiated cell lines had constitutive TCF activity [*P *< 0.046 (one-tailed), 0.071(two-tailed); Fisher Exact Probability Test]. This data supports the hypothesis that well-differentiated HCC cells display an autocrine/paracrine canonical Wnt signaling, probably because they co-express Wnt3 and several canonical Frizzled receptors. However, the great majority of poorly differentiated cell lines failed to generate canonical Wnt signaling activity, although they similarly co-expressed Wnt3 and canonical Frizzled receptors.

### Canonical Wnt signaling is repressed in poorly differentiated hepatocellular carcinoma cells

**Figure 9:**
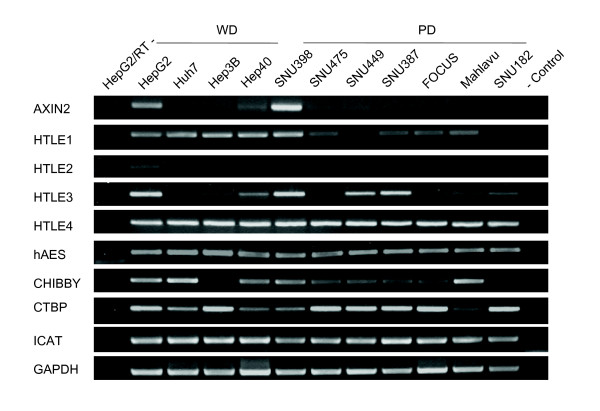
**Expression analysis of genes inhibiting canonical Wnt signaling downstream to β-catenin in HCC cells**. Total RNAs were extracted from cell lines and used to detect gene expression by RT-PCR assay. GAPDH RT-PCR was used as a loading control.

The lack of canonical Wnt activity in poorly differentiated cells could be due to either lack of sufficient Wnt ligand activity. Alternatively, canonical Wnt signaling could be repressed in these cell lines. A number of proteins downstream to β-catenin such as Axin2, HTLE family, hAES, Chibby, CTBP and ICAT are known to display inhibitory activity on canonical Wnt signaling [44]. We compared the expression of genes encoding these inhibitory proteins, but found no correlation with TCF activity or differentiation state (Figure 9).

**Figure 10:**
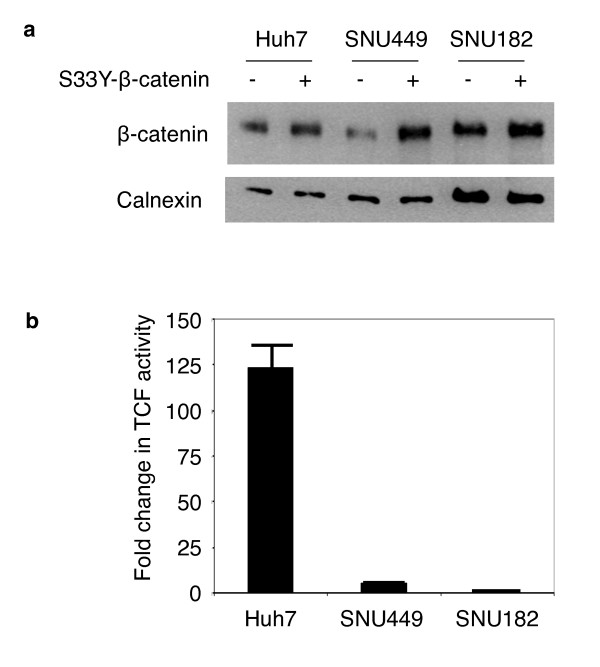
**Ectopic expression of mutant β-catenin induces high canonical Wnt activity in well-differentiated, but not in poorly differentiated hepatocellular carcinoma cells**. (**a**) Well-differentiated Huh7, and poorly differentiated SNU449 and SNU182 cell lines have been co-transfected with either pCI-neo-mutant β-catenin (S33Y) plasmid (S33Y-β-catenin +) or empty pCI-neo plasmid (S33Y-β-catenin -), and cellular β-catenin levels at post-transfection 48 h were tested by immunoblotting. Calnexin was used as a loading control. (**b**) Cell lines were treated as described, then pCI-neo-mutant β-catenin (S33Y)-transfected cells were subjected to TCF reporter assay. TCF activity denotes the ratio of signals detected with pGL3-OT (OT) and pGL3-OF (OF) plasmids, respectively. Assays in triplicate, error bars; SD. Co-transfections included pGL-OT or pGL-OF, in addition to pCI-neo plasmids in both (a) and (b).

Next, we compared TCF activity in Huh7, SNU449 and SNU182 cell lines following transient expression of a mutant (S33Y)-β-catenin (Figure 10). Transfection with S33Y-β-catenin resulted in an increase in total β-catenin protein in Huh7 and SNU449. This increase was less evident in SNU182 cells (Figure 10a). Well-differentiated Huh7 cells responded to S33Y-β-catenin expression by a strong activation of TCF/LEF reporter (130 folds). Under the same experimental conditions, the response of SNU449 cells was minimal (5 folds). More importantly, SNU182 cells were totally unresponsive (Figure 10b). These important differences between well-differentiated Huh7 and two different poorly differentiated cell lines (SNU449 and SNU182) are apparently not due to differences in transient transfection efficiencies, since the measured activities have been corrected for such differences (see material and methods section).

**Figure 11:**
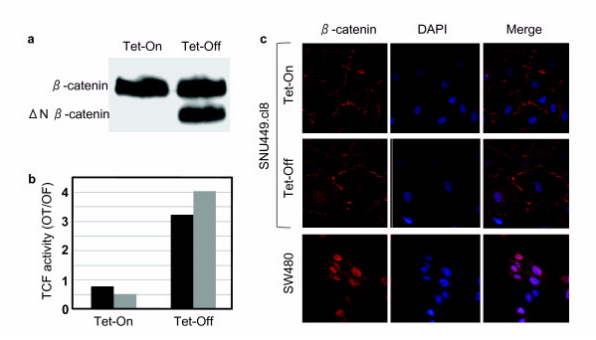
**Minimal TCF reporter activity and lack of nuclear accumulation of mutant β-catenin in poorly differentiated SNU449.cl8 cells**. SNU449 cells were stably transfected with Tet-responsive ΔN-β-catenin expression vector to obtain SNU448.cl8 cells. (**a**) Induced expression of N-terminally truncated ΔN-β-catenin protein in the Tet-Off conditions, as tested by western blot assay. Total cell lysates were extracted from cells and subjected to western blot assay using anti-β-catenin antibody. (**b**) TCF activity is only weakly induced in Tet-off conditions, as tested by duplicate experiments. (**c**) SNU449.cl8 cells at Tet-On state express wild-type endogenous β-catenin protein principally located at cell membrane. Under Tet-Off conditions the staining pattern remains almost identical despite ΔN-β-catenin expression. Note lack of nuclear accumulation. SW480 cells used as positive control display strong nuclear β-catenin staining. Cells were grown on coverslips, subjected to indirect immunofluorescence assay using anti-β-catenin antibody (red), counterstained with DAPI (blue) and examined by confocal microscopy.

In order to confirm the data on weakened response in poorly differentiated cell lines, we generated a clone from SNU449 cells (SNU449-cl8) with Tet repressor controlled expression of N-terminally truncated β-catenin (aa 98-781). N-terminally truncated β-catenin forms are frequently detected in cancer cells including HCC cells. They lack Ser/Thr phosphorylation sites (aa Ser23, Ser29, Ser33, Ser37, Thr41, Ser45) that are critically involved in its ubiquitin-mediated degradation, and they accumulate in the cell nucleus leading to oncogenic activation canonical Wnt signaling [27]. As shown in figure 11a, SNU449-cl8 cells expressed only wild-type β-catenin in the presence of tetracycline (Tet-on conditions), while expressing both wild-type and truncated β-catenin at comparable levels in Tet-off conditions. The induced expression of truncated β-catenin resulted in only a weak activation (3-4 fold) of TCF reporter activity, similar to data obtained by transient transfection experiments (Figure 11b). This low level of activation was similar to that seen in well-differentiated HCC cells in the absence of β-catenin or Axin-1 mutation (Fig. 8b), and strongly suggests that canonical Wnt signaling is actively repressed in this poorly differentiated HCC cell line. For comparison, well-differentiated HepG2 cells expressing wild-type and a similar N-terminally truncated β-catenin (Δ25-140 aa) activated TCF reporter gene by more than 60-fold (Figure 8a). In other words, although both Tet-off SNU449-cl8 and HepG2 cells displayed a heterozygous truncating β-catenin mutation, TCF activation was 15-fold less in poorly differentiated SNU449 background. To test whether this repression was related to cellular localization of β-catenin, we performed immunofluorescence detection using confocal microscopy (Figure 11c). Wild-type β-catenin was localized at the cell membrane with weak nuclear localization. The induction of truncated β-catenin in Tet-off SNU449-cl8 cells did not change this distribution significantly. We observed only weak cytoplasmic accumulation, with slight increases in both membrane and nuclear localization. In sharp contrast with these observations, in colorectal cancer cells (APC-mutated), used as a positive control [45], we detected strong nuclear accumulation of β-catenin by the same technique.

### WNT5A inhibits canonical Wnt signaling in HCC cells

**Figure 12:**
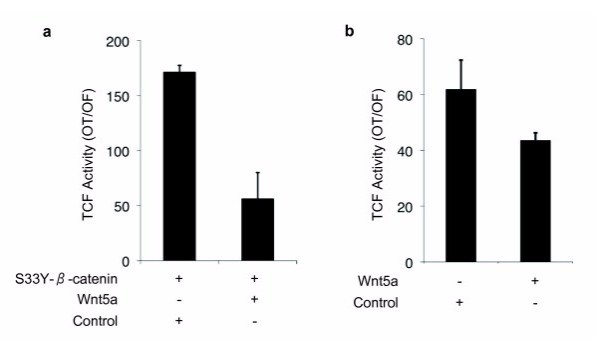
**Wnt5a inhibits canonical Wnt signaling activity in Huh7 and HepG2 cells**. (**a**) Huh7 cells were co-transfected with either pCI-neo-mutant β-catenin (S33Y) plasmid (S33Y-β-catenin +) along with pShuttle-IRES-WNT5a or empty pShuttle-IRES vector. 48 hours post transfection; cells were subjected to TCF reporter assay. TCF activity denotes the ratio of signals detected with pGL3-OT (OT) and pGL3-OF (OF) plasmids, respectively. Assays in triplicate, error bars; SD. Co-transfections included pGL-OT or pGL-OF, in addition to pCI-neo-S33Y-β-catenin and pIRES plasmids. (**b**) HepG2 experiments were performed under similar conditions, except that pCI-neo-mutant β-catenin (S33Y) plasmid was omitted.

Presently, the mechanism of repression of canonical Wnt signaling in poorly differentiated HCC cells is unknown, but it is associated with a lack of nuclear accumulation of β-catenin. Among noncanonical Wnt ligands, Wnt5a is best known for its antagonistic effect on canonical Wnt signaling [46]. Therefore, we tested the effect of ectopic Wnt5a expression on mutant-β-catenin-induced TCF activity in Huh7 cell line. In the absence of Wnt5a, TCF activity was induced more than 160-fold by mutant β-catenin in this cell line. Co-expression of Wnt5a resulted in three-fold repression of TCF activity (Figure 12a). To confirm our observations, we also tested the effects of Wnt5a on TCF activity induced by endogenous mutant β-catenin using HepG2 cell line. The expression of Wnt5a in this cell line caused a significant inhibition of TCF activation mediated by endogenous β-catenin (P < 0.05; Figure 12b). We concluded that Wnt5a that is selectively expressed in poorly differentiated HCC cell lines, and probably similarly acting noncanonical Wnt ligands are involved, at least partly, in the repression of canonical Wnt signaling in these cells.

## Discussion

Since the initial description of β-catenin mutations in HCCs in 1998 [33], Wnt signaling became a center of interest for these tumors. A large set of Wnt ligands and a large array of receptors are implicated in different cell processes by initiating canonical, but also noncanonical Wnt signals [35]. The antagonism between canonical and noncanonical Wnt pathways has also been reported [37,38,46]. Thus, both β-catenin and Wnt signaling are involved in highly complex cellular events of which only some are mediated by canonical Wnt pathway. This complexity is also observed during liver development and as well as in adult liver homeostatic events. Canonical Wnt signaling contributes to liver growth and regeneration, but also to liver "zonation" by controlling some liver-specific metabolic programs. ([47]). In addition, it contributes to the activation of liver stem or progenitor cells, as well as HCC-initiating cells [48-51].

Mutational activation of canonical Wnt signaling is not a frequent event in HCC, in contrast to hepatoblastoma displaying very high rates [52]. Mutations of β-catenin were restricted to a group of HCCs associated with low p53 mutation rate, negative HBV status and chromosomal stability, as stated earlier. These mutations were also associated with lower histological grade and better patient survival. Unexpectedly, β-catenin mutations are rare in more advanced and poorly differentiated HCCs [20,23]. Therefore, although considered to play an active role in HCC malignancy, the activation of canonical Wnt signaling may not be necessary for, or even repressed in advanced HCCs. Thus, β-catenin mutation and constitutive activation of canonical Wnt signaling may be differentiation-dependent events with mechanistic implications in HCC initiation and progression. We attempted to address this issue by using HCC-derived cell lines.

We first classified 11 HCC cell lines into "well-differentiated" and "poorly differentiated" subtypes using hepatocyte lineage, epithelial and mesenchymal cell markers, and in vitro migration assays. Well-differentiated HCC cell lines shared many features with hepatocytes such as expression of HNF-1α, HNF-4α, and E-cadherin, and epithelial morphology. Poorly differentiated cell lines were usually deficient in the expression of hepatocyte lineage and epithelial markers, but they expressed different mesenchymal markers strongly. These two types of HCC cell lines were also distinguished from each other by their in vitro behaviors. Poorly differentiated cell lines were usually more motile and more invasive than well-differentiated cell lines (Table 1). A global expression profiling study classified HCC cell lines in Group I and Group II [51]. Our well-differentiated and poorly differentiated cell line subtypes showed perfect correlation with Group I and Group II, respectively. Well-differentiated Group I was characterized by the activation of oncofetal promoters leading to increased expression of AFP and IGF-II, whereas poorly differentiated Group II was characterized by overexpression of genes involved in metastasis and invasion. Our well-differentiated and poorly differentiated subtypes were also in perfect correlation with respectively epithelial and mesenchymal HCC cell line types that have been identified very recently [12]. Mesenchymal cancer cells are considered as the products of EMT that is believed to be a key mechanism for the acquisition of invasive and metastatic capabilities by tumor cells [53]. Higher motility of poorly differentiated HCC cell lines reported here is in line with this concept. Thus, our two classes of cell lines share many similarities with well-differentiated and poorly differentiated HCC tumors. We used this model to compare the status of Wnt pathway according to HCC differentiation status.

A comprehensive analysis of Wnt signaling components in liver or hepatocytes is lacking. However, the expression of Wnt ligands and Frizzled receptors in mouse hepatocytes has been published [54]. Mouse hepatocytes expressed canonical receptors Fzd7 and Fzd9, as well as noncanonical Fzd2, Fzd3, Fzd4 and Fzd6. We observed highly similar pattern of expression in HCC cell lines with the exception of Fzd9 that showed weak expression. In addition, we detected increased expression of canonical Fzd1 and Fzd5 in most HCC cell lines. The expression frequency of these receptors was not associated with HCC cell differentiation status. Mouse hepatocytes expressed canonical Wnt1 and Wnt2, and noncanonical Wnt4, Wnt5a, Wnt5b and Wnt11. All or most HCC cell lines have lost the expression of canonical Wnt1 and Wnt2, but they displayed increased expression of canonical Wnt3 and Wnt10b ligands. Another canonical ligand, Wnt8b was expressed selectively in well-differentiated cell lines. In contrast, noncanonical Wnt4, Wnt5a and Wnt5b ligands were expressed in the majority of poorly differentiated cell lines, but not in most of well-differentiated cell lines. In addition, most HCC cell lines (poorly differentiated cell lines in particular) also displayed increased expression of noncanonical Wnt7b. Among Wnt ligands and Frizzled receptors that we found to be expressed or upregulated in HCC cell lines, Wnt3, Wnt4, Wnt5a, Fzd3, Fzd6 and Fzd7 have been previously reported to be overexpressed also in primary HCC tumors [55-57]. Overexpression of Wnt10b was also reported in HCC cels [58]. Increased levels of Wnt5a transcripts were detected in chronic hepatitis, cirrhosis and HCC [59]. A C-terminally mutated HBV X protein was shown to upregulate Wnt5a expression in HCC cells [60]. Thus, Wnt5a upregulation observed in clinical samples might be related to HBV at least in HBV-related liver diseases. Based on our observations that associate noncanonical Wnt ligand expression to poorly differentiated HCC cell lines, it will be interesting to test the predictive value of noncanonical Wnt expression for HCC prognosis.

Another important finding of this study is the differential activity of canonical Wnt signaling in different HCC subtypes. Well-differentiated cell lines displayed active canonical Wnt signaling at variable degrees. In addition to strong signaling activity associated to β-catenin and Axin1 mutations in two well differentiated cell lines, we also observed autocrine canonical Wnt signaling in two other well differentiated cell lines, as reported for some other cancer cell lines [36]. The functional significance of autocrine canonical Wnt signaling in these cell lines is not known.

However, small molecule antagonists of Tcf4/beta-catenin complex were shown to inhibit TCF reporter activity and down-regulate the endogenous Tcf4/β-catenin target genes c-Myc, cyclin D1, and survivin in Huh7 cells [61]. This observation strongly suggests that the autocrine canonical Wnt signaling is functional in well-differentiated HCC cell lines. Canonical Wnt signaling has been linked to both stem cell and cancer cell self-renewal in other cancer types. It was proposed that some adult cancers derive from stem/progenitor cells and that canonical Wnt signaling in stem and progenitor cells can be subverted in cancer cells to allow malignant proliferation [62]. Indeed, well-differentiated-HCC cell lines identified here such as HepG2, Huh7 and PLC/PRF/5 have been reported to harbor HCC stem cells [63]

Our third noteworthy observation was the lack of detectable canonical Wnt signaling activity in six out of seven poorly differentiated cell lines. Even a poorly differentiated cell line with a deleterious Axin1 mutation (SNU475) lacked detectable signaling activity. Thus, most probably, the canonical Wnt signaling was not only inactive, but also repressed in poorly differentiated HCC cell lines. In confirmation of this expectation, transient or Tet-regulated expression of mutant β-catenin failed to generate significant canonical Wnt signaling activity in two different poorly differentiated cell lines. Furthermore, we linked this weak activity to poor nuclear accumulation of β-catenin protein in SNU449.cl8 cell line. Thus, unlike well-differentiated cell lines, poorly differentiated HCC cells displayed strong resistance to canonical Wnt signal activation.

The mechanisms of resistance to canonical Wnt signal activation in poorly differentiated HCC cells are presently unknown. We provide here one potential mechanism. Wnt5a has been previously implicated in canonical Wnt signaling as an antagonist and regulator of β-catenin levels in other cell types [48,49]. Using both ectopic and endogenous mutant β-catenin expression systems in two different cell lines, we demonstrated that co-transfections with Wnt5a-expressing plasmid can significantly inhibit canonical Wnt signaling in HCC cells. The mechanism of Wnt5a antagonism on canonical Wnt signaling in HCC cells is not known. In breast cancer cells, the loss of Wnt5a signaling resulted in stabilization of nuclear beta-catenin and expression of Wnt/beta-catenin target genes [64]. However, both ectopically and endogenously expressed mutant β-catenins used in our experiments were N-terminally truncated devoid of their Ser/Thr phosphorylation motifs. Thus, Wnt5a appears to inhibit canonical Wnt signaling in HCC cells, downstream to β-catenin, independent of its glycogen synthase 3-β- and bTrCO-dependent degradation. Wnt5a has been shown to inhibit canonical Wnt signaling either by bTrCP-independent proteasomal degradation [65], or, by downregulating β-catenin-induced reporter gene expression without influencing β-catenin levels [37] in kidney epithelial cells. Wnt5a may use similar mechanisms in Huh7 and HepG2 cells. Further studies with downregulation of noncanonical Wnt ligands in poorly differentiated HCC cell lines may help to better define the implications of such ligands in liver cancer biology.

The role of Wnt5a in cancer is complex. It may play tumor-promoting or tumor-suppressing functions depending on cellular context. Wnt5a has been described as a tumor promoter in melanoma, gastric, pancreas, prostate cancer, but as a tumor suppressor in HCC, neuroblastoma, leukemia, colon, and thyroid cancers [46]. The inability of Wnt5a to transform cells or signal through the canonical β-catenin pathway pointed that it cannot promote tumorigenesis by upregulation of canonical Wnt signaling, unlike canonical Wnt ligands [66]. Our results suggest that Wnt5a, upregulated in poorly differentiated highly motile mesenchymal-like HCC cells may play a role in tumor progression by inducing EMT. Upregulation of Wnt5a expression during EMT has been reported [67]. Furthermore, the Wnt5A/Protein kinase C pathway was shown to mediate motility in melanoma cells via of an EMT [68]. Similarly, CUTL1-upregulated Wnt5a significantly enhanced migration, proliferation and invasiveness in pancreas cancer cellls [69]. These effects were accompanied by a marked modulation of marker genes associated with EMT. Wnt5a may promote EMT in HCC cells by a similar mechanism.

The expression status of Wnt5a in HCC is not well known. To our knowledge, only one report addressed this issue [59]. Compared to normal tissue, Wnt5a mRNA expression was strongly induced in HCC, as well as in chronic hepatitis and cirrhosis. However, immunostaining of Wnt5a protein showed a bell-shaped pattern: low to undetectable levels were present in normal tissue and in tumor samples, whereas strong immunostaining was seen in chronic hepatitis, cirrhosis and dysplastic liver cells. The reasons of the discrepancy between transcript and protein expression in HCC tissues are not known presently. However, it appears that peritumoral liver tissues express high levels of Wnt5a protein that could trigger noncanonical Wnt signaling in adjacent tumor cells. It will be important to further investigate the role of Wnt5a in HCC tumor progression.

Taken together, our studies demonstrate that canonical Wnt activity is active in well-differentiated, but repressed in poorly differentiated HCC cell lines. This correlates with in vivo tumor studies indicating that β-catenin mutations are prevelant in well-differentiated, but not in poorly differentiated tumors. In addition, we showed that poorly differentiated cell lines express noncanonical Wnt ligands such as Wnt5a acting as an antagonist of canonical Wnt signaling. Thus, it appears that HCC cells may activate or repress their canonical Wnt signaling, using autocrine/paracrine systems based on selective use of canonical and noncanonical Wnt ligands.

We hypothesize that the active canonical Wnt signaling observed in well-differentiated HCC cells contributes to tumor initiation, but not necessarily to tumor progression. Instead, noncanonical Wnt signaling may be used by poorly differentiated HCC tumors to promote cell motility and invasion. Selective use of canonical and noncanonical Wnt signaling at different stages may be a key mechanism involved in hepatocellular carcinogenesis. Recent studies showed that canonical Wnt signaling contributes to the self-renewal and expansion of HCC-initiating cells with stem/progenitor cell features [50,70]. However, the lack of HCC development in β-catenin transgenic mice strongly suggests that canonical Wnt signaling activation has limited tumorigenic potential in liver tissue. Indeed, recent studies showed that canonical Wnt signaling plays a major role in the specification of mature hepatocytes for perivenous-specific gene expression ([71,72]. Such a hepatocyte differentiation function of canonical Wnt signaling may not be compatible with cellular dedifferentiation that goes along with HCC development. Therefore, alternative pathways such as Wnt5a-mediated noncanonical Wnt signaling may be necessary for sustained growth and progression of HCC tumors. Melanoma may serve as a demonstrated model to our hypothesis. Similar to HCC, canonical Wnt signaling activation is an early event and nuclear β-catenin accumulation is associated with better patient survival in melanoma. Nuclear β-catenin is lost in more aggressive melanomas that express Wnt5a that promotes EMT, cell motility and metastasis[46]. Chien et al. [73] have recently demonstrated that canonical Wnt signaling induces growth inhibition and differentiation in melanoma cells, whereas Wnt5a can antagonize some of these effects. These findings clearly establish a dual function of Wnt signaling in melanoma. In light of these recent developments, our findings call for further investigations on respective roles of canonical and noncanonical Wnt signaling in HCC.

## Conclusion

Our observations support the hypothesis that Wnt pathway is selectively activated or repressed depending on differentiation status of HCC cells. We propose that canonical and noncanonical Wnt pathways have complementary roles in HCC, where the canonical signaling contributes to tumor initiation, and noncanonical signaling to tumor progression.

## Methods

### Cell lines

Hepatocellular carcinoma cell lines Huh7, Hep40, Hep3B, Hep3B-TR, FOCUS, Mahlavu, SNU182, SNU 387, SNU 398, SNU423, SNU 449, SNU 475, PLC/PRF/5, SK-Hep-1, hepatoblastoma cell line HepG2 and colorectal cancer cell line SW480 were cultivated as described previously [41].

### Reverse transcription-polymerase chain reaction (RT-PCR) analysis

Total RNAs were extracted from cultured cells using NucleoSpin RNA II Kit (MN Macherey-Nagel, Duren, Germany) according to the manufacturer's protocol. The cDNAs were prepared from total RNA (2 μg) using RevertAid First Strand cDNA Synthesis Kit (MBI-Fermentas, Vilnius, Lithuania). A negative control without reverse transcriptase (1 μl ddH2O instead) was also prepared for each sample. All PCR reactions were carried out using 1 μl cDNA from the reverse transcription mix, for 35 cycles except GAPDH, which was amplified for 24 cycles. Negative controls without reverse transcriptase were included for each set of primers (primer sequence information is available upon request). PCR products were analyzed on a 2% (w/v) agarose gel.

### Wound-healing assay

Cells were cultured in six-well culture plates in RPMI 1640 or DMEM with 10% FBS. A single linear wound was made with a p200 pipette tip in confluent monolayer cells. The distances between wound edges were measured at fixed points in each dish according to standardized template. Debris were removed by washing the cells twice with PBS and then cells were incubated in RPMI 1640 or DMEM with 2% FBS. After 24 hours migration, cells were fixed with methanol and stained with 0.2% crystal violet. Cell migration into the wound was visualized using phase contrast microscopy (x20 magnification). The number of cells migrating beyond the wound edge was quantified microscopically in the randomly selected fields for each triplicate well.

### Immunocytochemistry

Cells were grown on coverslips, fixed in 4% formaldehyde, permeated with 0.5% saponin/0.1% Triton X-100, and stained with mouse monoclonal anti-human vimentin antibody (Dako) using Envision kit (Dako), developed with diaminobenzidine, and counterstained with hematoxylin.

### Confocal microscopy

Cells were grown on slides in 6 well plates and were fixed in 3.5% paraformaldehyde (PFA) for 15 minutes, and permealized using 0,25% Triton-X-100 for 10 min. Non specific protein binding was blocked by 30 minutes of incubation with 5% bovine serum albumin (BSA) in phosphate-buffered saline (PBS) at room temperature. Cells were then incubated 2 hours with monoclonal anti-β-catenin antibody M5.2 (1:200 dilution) in 1% BSA in PBS at room temperature in a moist chamber. Immunofluorescence staining was obtained by incubating for 1 hour with Alexa Fluor^® ^594 F(ab')2 fragment of rabbit anti-mouse IgG (H+L) (Invitrogen) (dilution 1:750). Cells were counterstained with DAPI (dilution 1:750), slides were mounted using ProLong^® ^Gold antifade reagent (Invitrogen) and examined under Zeiss LSM 510 Meta laser scanning confocal microscope (MPI Freibourg, Germany) using 488 nm and 543 nm laser excitation lines, and photographed.

### Plasmids

The pShuttle-IRES-Wnt5a expression plasmid was constructed by subcloning of an EcoRI-cut Wnt5a cDNA fragment from plasmid pGEMTz-Wnt5a vector (a gift from R. Kemler) into BglII site of the pShuttle-IRES-hrGFP-1 vector (Stratagene, USA). pCI-Neo-mutant β-catenin (S33Y) expression plasmid, and pGL3-OT and pGL3-OF reporter plasmids were kindly provided by B. Vogelstein. Other plasmids were pCI-Neo (Promega) and pEGFP-N2 (Clontech, Palo Alto, CA). The pAUCT-ΔN-β-catenin plasmid expressing N-terminally truncated β-catenin (aa 98-781) under the control of Tet repressor was constructed using pAUCT-CCW vector (gift from Ali Fattaey, USA). A cDNA fragment of XhoI-NotI digestion from pCI-Neo-mutant β-catenin (S33Y) plasmid was inserted into XhoI-NotI site of pAUCT-CCW vector.

### Transfections

Endogenous TCF/LEF-dependent transcriptional activity was tested by using pGL3-OT and pGL3-OF reporter plasmids, as described previously [41], except that cells were transfected using Lipofectamin 2000 reagent (Invitrogen), following instructions provide by the supplier. Mutant β-catenin-induced TCF/LEF-dependent transcriptional activity was tested after co-transfection of cells with pCI-Neo-mutant β-catenin (S33Y) expression plasmid (1.75 μg/well) together with the reporter plasmids. pCI-Neo (1.75 μg/well) was used as negative control. The effect of Wnt5a expression on TCF/LEF-dependent transcriptional activity was tested using Huh7 and HepG2 cell lines. Huh7 cell line was co-transfected with pCI-Neo-mutant β-catenin (S33Y) expression plasmid (1 μg/well) together with either pShuttle-IRES-Wnt5a (0.75 μg/well) or the empty vector pShuttle-IRES-hrGFP-1 (0.75 μg/well) and pGL3-OT/pGL3-OF reporter plasmids (0.75 μg/well for each). At 48 h following transfection, luciferase assay was performed by using Luciferase Reporter Gene Assay, constant light signal kit (Roche Diagnostics GmbH., Mannheim, Germany). Luciferase activity was read with The Reporter^® ^Microplate Luminometer (Turner BioSystems Inc., Sunnyvale, CA) and data was normalized according to transfection efficiency obtained with each transfection, as described previously [41]. HepG2 cell line was co-transfected with either pShuttle-IRES-Wnt5a (0.5 μg/well) or the empty vector pShuttle-IRES-hrGFP-1 (0.5 μg/well) and pGL3-OT/pGL3-OF reporter plasmids (0.5 μg/well for each), with an internal control (0.05 μg/well pRL-TK Renilla luciferase vector) in a 12-well plate, using Lipofectamine 2000 Transfection Reagent. Forty-eight hours post-transfection, the cells were washed with PBS, and lysed in passive lysis buffer (Dual Luciferase kit; Promega). The cell lysates were transferred into an OptiPlate 96-well plate (Perkin-Elmer) and assayed in a 1420-Multilabel counter luminometer, VICTOR3 (Perkin-Elmer) using the Dual-Luciferase kit (Promega). Relative TOP-FLASH luciferase units were measured and normalized against Renilla luciferase activity and further normalized luciferase activity against FOP-FLASH activity. All transfection experiments were performed in triplicate and data were expressed as mean of triplicate values (±S. D.) and p values were calculated. TCF/LEF activity was reported as the ratio of normalized luciferase activities obtained with pGL-OT and pGL-OF plasmids, respectively (mean ± S. D.).

### Generation of mutant β-catenin expressing SNU449-cl8 cell line

SNU449 cell line clone ectopically expressing N-terminally truncated (aa 98-781) β-catenin, under the control of Tet repressor was generated by stable transfection with pAUCT-ΔN-β-catenin plasmid. Briefly, SNU449 cells were plated onto 6-well plate and transfected with 2 μg of plasmid DNA using Lipofectamin 2000 reagent (Invitrogen). 24 hours post transfection, cells were transferred to 90 mm dishes and subjected to G418 (0.6 μg/ml) selection in the presence of tetracycline (1 μg/ml) until resistant cell colonies became visible. Several clones were tested by Western blot for the expression of N-terminally truncated (aa 98-781) β-catenin after withdrawing tetracycline from the culture medium to induce the expression of the transgene. Only one clone (SNU449-cl8) displayed Tet-dependent expression of mutant β-catenin, and it was used for further studies.

### Western blotting

Detergent-soluble cell lysates were prepared at 48 h post-transfection and used for western blot analysis, as described previously [41]. Antibodies to β-catenin (Santa Cruz Biotechnology, Inc., CA) and calnexin (Sigma) were obtained commercially. ECL kit (Amersham Life Science, Inc., Piscataway, NJ) was used for detection of antigen-antibody complexes. Equal protein loading was verified by Western blot assay with calnexin antibody.

### Statistical analysis

The statistical significance of the active TCF reporter activity between well-differentiated and poorly differentiated HCC cell lines was tested by Fisher Exact Probability Test using an on-line tool .

## List of abbreviations

AFP: α-fetoprotein; EMT: epithelial to mesenchymal transition; FZD: Frizzled; HCC: hepatocellular carcinoma; HBV: hepatitis B virus; HNF: hepatocyte nuclear factor; PD: poorly differentiated; RT-PCR: reverse transcriptase-polymerase chain reaction; WD: well differentiated

## Competing interests

The authors declare that they have no competing interests.

## Authors' contributions

HY, KB, SS and EE carried our RT-PCR analyses. HY carried out confocal microscopy and western blot analyses. SS and MY carried out the immunocytochemistry. HY, HB, NO and NT carried out TCF reporter activity assays. KB constructed SNU440.cl8 cell line. EC, AT and NA carried out wound-healing and cell migration assays. KCA contributed to the design of the study. M.O. conceived of, designed and coordinated the study. MO., HY and KB drafted the manuscript. All authors read and approved the final manuscript.

## Authors' information

Nuri Ozturk - present address: Department of Biochemistry and Biophysics, University of North Carolina School of Medicine, Chapel Hill, North Carolina 27599. USA.

Nilgun Tasdemir - present address: Cold Spring Harbor Laboratory, One Bungtown Road, Cold Spring Harbor, NY 11724, USA.
